# Childhood Brain Tumors, Residential Insecticide Exposure, and Pesticide Metabolism Genes

**DOI:** 10.1289/ehp.0901226

**Published:** 2009-10-05

**Authors:** Susan Searles Nielsen, Roberta McKean-Cowdin, Federico M. Farin, Elizabeth A. Holly, Susan Preston-Martin, Beth A. Mueller

**Affiliations:** 1 Public Health Sciences Division, Fred Hutchinson Cancer Research Center, Seattle, Washington, USA;; 2 Norris Comprehensive Cancer Center/Department of Preventive Medicine, Keck School of Medicine, University of Southern California, Los Angeles, California, USA;; 3 Center for Ecogenetics and Environmental Health, Department of Environmental and Occupational Health Sciences, University of Washington, Seattle, Washington, USA;; 4 Department of Epidemiology and Biostatistics, School of Medicine, University of California, San Francisco, San Francisco, California, USA;; 5 Department of Epidemiology, School of Public Health and Community Medicine, University of Washington, Seattle, Washington, USA

**Keywords:** acetylcholinesterase inhibition, childhood cancer, children, gene–environment interaction, insecticides, pesticides, xenobiotic metabolism

## Abstract

**Background:**

Insecticides that target the nervous system may play a role in the development of childhood brain tumors (CBTs). Constitutive genetic variation affects metabolism of these chemicals.

**Methods:**

We analyzed population-based case–control data to examine whether CBT is associated with the functional genetic polymorphisms *PON1*_C–108T_, *PON1*_Q192R_, *PON1*_L55M_, *BCHE*_A539T_, *FMO1*_C–9536A_, *FMO3*_E158K_, *ALDH3A1*_S134A_, and *GSTT1* (null). DNA was obtained from newborn screening archives for 201 cases and 285 controls, ≤ 10 years of age, and born in California or Washington State between 1978 and 1990. Conception-to-diagnosis home insecticide treatment history was ascertained by interview.

**Results:**

We observed no biologically plausible main effects for any of the metabolic polymorphisms with CBT risk. However, we observed strong interactions between genotype and insecticide exposure during childhood. Among exposed children, CBT risk increased per *PON1*_–108T_ allele [odds ratio (OR) = 1.8; 95% confidence interval (CI), 1.1–3.0] and *FMO1*_–9536A_ (*6) allele (OR = 2.7; 95% CI, 1.2–5.9), whereas among children never exposed, CBT risk was not increased (*PON1*: OR = 0.7; 95% CI, 0.5–1.0, interaction *p* = 0.005; *FMO1*: OR = 1.0; 95% CI, 0.6–1.6, interaction *p* = 0.009). We observed a similar but statistically nonsignificant interaction between childhood exposure and *BCHE*_A539T_ (interaction *p* = 0.08). These interactions were present among both Hispanic and non-Hispanic white children.

**Conclusion:**

Based on known effects of these variants, these results suggest that exposure in childhood to organophosphorus and perhaps to carbamate insecticides in combination with a reduced ability to detoxify them may be associated with CBT. Confirmation in other studies is required.

Both environmental exposure and genes affect childhood brain tumor (CBT) development. Ionizing radiation to the head and selected heritable syndromes are established risk factors (Fisher et al. 2007). However, these account for only a small proportion of CBT cases. Several epidemiologic studies suggest pesticides might be associated with CBT (Infante-Rivard and Weichenthal 2007), but most relied on retrospective questionnaire data with little detail. One study that considered the type and timing of pesticide exposure observed an increased risk of CBT with prenatal exposure to flea/tick products, but not herbicides, fungicides, or molluscicides (Pogoda and Preston-Martin 1997). The specificity of this finding is interesting because the major classes of insecticides—organophosphorus (OP), carbamate, organochlorine (OC), and pyrethrin/pyrethroid—readily cross the blood–brain barrier and target the nervous system, whereas pesticides aimed at plants and fungi inherently rely on different mechanisms of action.

Because constitutive genetic variation influences insecticide metabolism, we previously examined whether CBT is associated with two single nucleotide polymorphisms (SNPs) in the gene that codes for paraoxonase (PON1) (Searles Nielsen et al. 2005). We observed no association between CBT and the single coding region SNP *PON1*_Q192R_ but a strong dose–response relationship between CBT and *PON1*_C–108T_, a promoter-region SNP associated with enzyme levels. CBT risk was increased among children who presumably had a reduced ability to detoxify chlorpyrifos and diazinon, the most common residential insecticides for many years, and this association was restricted to children whose homes had been chemically treated for insects. Here we examine these SNPs and six additional genetic polymorphisms that affect insecticide metabolism using an expanded number of cases and controls born in Washington State and California.

## Materials and Methods

### Participant selection and specimen retrieval

This analysis includes children enrolled in the U.S. West Coast CBT Study, a population-based case–control study described previously (Preston-Martin et al. 1996). Briefly, those cases were children diagnosed from 1984 through 1991 with a primary tumor of the brain, cranial nerves, or meninges [*International Classification of Diseases–Oncology* (ICD-O) (World Health Organization 1976) codes 191.0–192.1] and who were identified through the Surveillance, Epidemiology and End Results registries in the Seattle–Puget Sound region of Washington, the San Francisco–Oakland area of California, and Los Angeles County, California. Control children living in the same counties were identified by random digit dialing. Children with a biological mother who spoke English or Spanish in a home with a telephone were eligible. After informed consent, interviews were completed with the mothers of 75% of controls who met these criteria (88% screened) and who were invited to participate while being frequency matched to cases by age and sex (1:1 in Los Angeles and 2:1 elsewhere) and 73% of cases for whom physician permission was received (97% of all cases) and who met the above criteria (or potentially met them but who were not located, 13% of all cases).

We identified those participating children for whom a dried blood spot (DBS) from newborn screening might still be archived at the Washington State Department of Health (born in Washington in 1978–1990) or the California Department of Public Health (born in California in 1982–1990). As detailed elsewhere (Searles Nielsen et al. 2008), DBS were located and anonymized for 88% of children for whom a specimen was sought in Washington, including 66 (94%) cases and 137 (86%) controls included in both the present and earlier (Searles Nielsen et al. 2005) CBT–*PON1* analyses. Similar specimen collection methods were used in California, where DBS for 86% of sought children were located, including 26 (93%) cases and 50 (75%) controls from San Francisco and 110 (92%) cases and 99 (85%) controls from Los Angeles. Institutional review board approvals from all relevant agencies were obtained before the study began.

### Genotyping

The Functional Genomics Core Laboratory of the Center for Ecogenetics and Environmental Health at the University of Washington in Seattle, which was unaware of case status, obtained DNA using commercially available kits [QIAamp DNA Mini Kit for Washington DBS (Searles Nielsen et al. 2005) and the Repli-g Kit (Paynter et al. 2006) for California DBS; Qiagen, Valencia, CA], determined glutathione *S*-transferase theta 1 (GSTT1) null status (Kelada et al. 2003), and used custom TaqMan Detection System–based assays-by-Design service (Applied Biosystems, Inc., Foster City, CA) for seven functional pesticide metabolism SNPs (Table 1) and *PON2*_S311C_ (rs7493). We included *PON2*_S311C_ to investigate whether CBT–*PON1* associations might be a result of PON1’s generic antioxidant capabilities, because PON2 does not metabolize OPs (Draganov et al. 2005). Fragment lengths required for the TaqMan assays ranged from 84 to 202 bp (base pairs) and from 215 to 480 bp for the *GSTT1* assay. Negative controls (no DNA) and sequencing-verified positive controls were included in each batch of analyses. Results were verified by sequencing as needed, and approximately 10% of all samples were reassayed. For the Washington samples, blind duplicate or quadruplicate specimens for 6% of cases and 6% of controls were assayed for all nine polymorphisms [205 of 207 (99.0%) pairs agreed], and the *PON1*_C–108T_ assay results were confirmed using a different TaqMan assay (100% concordance). Complete genotyping data were available for all but two cases and one control.

### Insecticide exposure assessment

During a structured in-person interview, children’s mothers reported whether they or anyone else had chemically treated the child’s home for pests listed on a show card that included termites, fleas, ants, cockroaches, silverfish, or “other” pests. The questionnaire inquired separately about treatments during pregnancy (1 month before conception until birth) and childhood (birth until diagnosis for cases or comparable reference date for controls). We considered these distinct periods of exposure separately in our analyses because *a*) the dose of the active ingredients and their intermediates (e.g., oxons) may be altered by maternal enzymes when the child is *in utero; b*) the child’s enzyme levels [those shown in Table 1 and oxon-forming cytochrome P450 (CYP) isoforms] vary developmentally—some in direct response to birth; and *c*) timing of insecticide exposure appears important for CBT (Pogoda and Preston-Martin 1997). These data were available for all but one case and one control with DBS and were ascertained an average of 5.3 and 6.4 years, respectively, after birth.

Supplementary data collected in a follow-up study in Los Angeles (Pogoda and Preston-Martin 1997) allowed us also to consider exposure to any residential insecticides (treatment of the home, yard, garden, pets, and/or for lice) and exposure to the most common specific treatments (for fleas/ticks, for nuisance insects) among 80 (40%) cases and 68 (24%) controls in the present work (73% of cases and 69% of controls from Los Angeles and with DBS).

### Statistical analysis

We used unconditional logistic regression to compute odds ratios (OR) and 95% confidence intervals (CIs) of CBT in relation to each polymorphism. The *GSTT1* assay provided dichotomous (any/no GSTT1) results. For all other polymorphisms we checked Hardy–Weinberg equilibrium by exact chi-square test [butyrylcholinesterase (*BCHE*) and flavin-containing monooxygenase 1 (*FMO1*) SNPs, with a minor allele frequency of ≤ 0.20] or Pearson’s chi-square test (other SNPs) and, unless noted, modeled genotype linearly (coded the variable as 0, 1, or 2 hypothesized “high-risk” alleles) (Table 1). Likelihood ratio tests confirmed that single linear variables were appropriate. We adjusted all models for study center, sex, diagnosis/reference age, and race/ethnicity [African American (either parent African American), Hispanic (not African American, and either parent Hispanic), white (both parents non-Hispanic white), Asian/other]. We excluded five cases and two controls with unknown race/ethnicity.

We conducted haplotype analyses for the four PON SNPs and the two FMO SNPs. We inferred haplotypes (PHASE software, version 2.1; Stephens and Donnelly 2003) while accounting for distance between SNPs, and using 100 additional control DBS (anonymous children born in Washington in 1980–1991). We modeled haplotype linearly while including children for whom both alleles were sufficiently frequent (> 1%) and estimated with > 80% probability.

To examine the potential for gene–insecticide interaction, we estimated separate CBT-genotype ORs for *a*) unexposed children (never exposed during pregnancy or childhood), *b*) children exposed during pregnancy, and *c*) children exposed during childhood. We formally assessed interaction on a multiplicative scale in logistic regression; reported *p*-values are from a single product term (genotype multiplied by dichotomous exposure). Prenatal and childhood insecticide exposure were correlated, so, when possible, we stratified prenatal exposure models by childhood exposure, and vice versa. We attempted to confirm all observed interactions in case-only gene–environment models (Khoury and Flanders 1996), because these would not be influenced by the composition of our control group or their reporting of insecticide use.

We checked whether our main gene and gene–insecticide CBT ORs were consistent across racial/ethnic groups, age, and study center. For these comparisons we dichotomized diagnosis/reference age at the median (3 years), combined centers (California, Washington), and examined stratum-specific estimates for sufficiently large racial/ethnic groups (Hispanic, non-Hispanic white). We also considered CBT histologic subtype. Included among the present sample were ICD-O histology codes 9380, 9382, 9400, 9401, 9420, 9421 (astroglial tumors, *n* = 96, 48% cases), 9470, 9471, 9473 (medulloblastoma/primitive neuroectodermal tumors, *n* = 55, 27% cases), and 9391–9393 (ependymoma, *n* = 25, 12% cases).

## Results

All children were ≤ 10 years of age at diagnosis/reference, and most were < 5 years of age (Table 2). Proportionally more cases than controls were Hispanic or nonwhite. Only three cases and three controls had a heritable syndrome that predisposes to brain tumor, or a first-degree relative with a history of brain tumor. Farm residence and maternal prenatal agricultural occupation also were uncommon (2–4% cases, 1–2% controls).

### Residential insecticide exposure

During pregnancy, proportionally more mothers of cases (27%) than controls (21%) reported treatment of the home for termites, fleas, ants, cockroaches, silverfish, or other pests (Table 2); we did not observe this difference in Washington State, where treatment was less prevalent than in California (data not shown). In contrast, treatment of the home for insects during childhood was more common among controls (33%) than among cases (23%) (Table 2), a difference observed in all study centers (data not shown). Among children in the pesticide follow-up study in Los Angeles, any residential insecticide use was prevalent both during pregnancy and childhood (≥ 70% of cases and controls). In general, use of flea/tick products was more common among cases than among controls, and the reverse for nuisance pests.

### CBT and pesticide metabolism polymorphisms

Genotype frequencies were in Hardy–Weinberg equilibrium for each racial/ethnic group (all *p*-values > 0.05). Overall, we observed no marked differences between cases and controls for any polymorphism [see Supplemental Material, Table 1 (available online at doi:10.1289/ehp.0901226.S1 via http://dx.doi.org)]. Any potential heterogeneity in the CBT–genotype ORs by race/ethnicity was not statistically significant (all interaction *p*-values > 0.24). Main effect ORs for all racial/ethnic groups combined (and adjusted for this factor) were close to null, with the possible exception of *BCHE*_A539T_ and *FMO3*_E158K_ (Table 3). FMO haplotype analyses suggested that any increased CBT risk in relation to the *FMO3*_158K_ allele was restricted to children with two *FMO1*_–9536C_ alleles, but 95% CIs were wide (data not shown).

### Genotype–insecticide interactions

We observed statistically significant interactions between insecticide treatment of the home during childhood and two promoter region pesticide metabolism SNPs (interaction *p* = 0.005 for *PON1*_C–108T_, 0.009 for *FMO1*_C–9536A_; Table 3). We also observed an interaction for the coding region SNP *BCHE*_A539T_ of borderline statistical significance (interaction *p* = 0.08). ORs per “high-risk” (hypothesized poor detoxification) allele (*PON1*_–108T_, *FMO1*_–9536A_, and *BCHE*_539T_) were greater among children whose homes had been treated during childhood than among children whose homes never had been treated. These interactions were present among non-Hispanic white children (interaction *p* = 0.11 for *PON1*_C–108T_, 0.04 for *FMO1*_C–9536A_, 0.04 for *BCHE*_A539T_) and Hispanic children (interaction *p* = 0.13 for *PON1*_C–108T_, 0.12 for *FMO1*_C–9536A_, 0.16 for *BCHE*_A539T_; Table 4). We observed the interactions between childhood insecticide exposure and *PON1*_C–108T_ and *FMO1*_C–9536A_ with or without exposure during pregnancy [all interaction *p* = 0.01–0.06; see Supplemental Material, Table 2 (doi:10.1289/ehp.0901226.S1)]. These interactions also appeared independent of nearby SNPs, because we observed the insecticide–*FMO1*_C–9536A_ interaction across *FMO3*_E158K_ genotypes, and the insecticide–*PON1*_C–108T_ interaction when modeling *PON1*–*PON2* as a haplotype. The *PON1*_C–108T_ interaction appeared to vary by age at diagnosis/reference: Among children < 3 years of age at diagnosis/reference, the OR per *PON1*_–108T_ allele was 2.4 (95% CI, 1.0–5.7) if exposed to insecticides and 0.5 (95% CI, 0.3–0.7) if unexposed (interaction *p* = 0.001), and among older children 1.4 (95% CI, 0.7–2.7) if exposed and 1.2 (95% CI, 0.7–2.0) if unexposed (interaction *p* = 0.69; data not shown). This did not appear to be a result of the correlation between age and birth year or between prenatal and childhood exposure.

When we stratified genotype ORs by home insecticide treatment during pregnancy, we observed variability between exposed and unexposed children for some of the pesticide metabolism polymorphisms (Table 3). For example, the *GSTT1* null genotype was associated with a reduced risk of CBT only among the exposed children, and the “high-risk” *FMO3*_158K_ allele was associated with an increased risk of CBT only among the unexposed children. However, none of the possible interactions between genotype and prenatal insecticide exposure was statistically significant (each interaction *p* > 0.15). Statistically significant or borderline interactions were suggested only in modestly sized subgroups involving *PON1*_C–108T_ and *FMO1*_C–9536A_ (data not shown).

Among children from Los Angeles with supplementary pesticide data, we observed possible interactions between both prenatal and childhood insecticide exposure and *BCHE*_A539T_ (genotype dichotomized; interaction *p* = 0.05–0.06 for any residential insecticides, 0.05–0.13 for flea/tick products, and 0.06–0.16 for products for nuisance pests such as ants and cockroaches; data not shown). The “high-risk” *BCHE*_539T_ allele was associated with increased CBT risk only among insecticide-exposed individuals.

Even within the larger sample, our ability to consider histologic tumor type was quite limited. Nonetheless, the interactions between insecticide treatment of the home during childhood and each of the three SNPs (*PON1*_C–108T_, *FMO1*_C–9536A_, and *BCHE*_A539T_) remained when we focused on our largest subgroup, astroglial tumors. However, these interactions were not strictly specific to this tumor type.

We were unable to formally confirm any interactions using case-only models because, among controls, genotype and exposure were not independent. Otherwise, these models supported all reported interactions.

## Discussion

We attempted to build on prior studies of CBT and pesticide exposure by considering individual differences in the metabolism of insecticides that target the nervous system. We *a priori* designated a “high-risk” allele for each polymorphism based on the expected functional impact with respect to acetylcholinesterase (AChE) inhibition (OP and carbamate insecticides; *PON1*, *BCHE*, *FMO1*, *FMO3*, and *GSTT1* polymorphisms) and ion channel stimulation [OC and pyrethroid insecticides; aldehyde dehydrogenase 3A1 (*ALDH3A1*) and *GSTT1* polymorphisms]. Although some insecticides metabolized by these enzymes are ubiquitous in the environment or diet, ORs for CBT in relation to the hypothesized “high-risk” allele for the nine polymorphisms were close to the null, or fluctuated equally above and below the null. However, we observed interactions between genotype and chemical treatment of the home for insects during childhood for three functional SNPs located on different chromosomes: *PON1*_C–108T_, *FMO1*_C–9536A_, and *BCHE*_A539T_. The direction of these interactions was consistent and biologically plausible. Moreover, they were present in each of our two largest racial/ethnic groups.

*PON1*_–108T_ and *BCHE*_539T_ variants are respectively associated with reduced *in vivo* activity of PON1 (Brophy et al. 2001; Chen et al. 2003; Leviev and James 2000) and the butyrylcholinesterase enzyme (BuChE) (Babaoglu et al. 2004; Bartels et al. 1992; Maetzler et al. 2009). Both neutralize AChE inhibitors: PON1 hydrolyzes selected OPs, notably chlorpyrifos and diazinon (Furlong 2007), and BuChE sequesters all OP and carbamate insecticides (Cokuğraş 2003). *In vitro* studies suggest that *FMO1*_–9536A_ materially reduces promoter activity (Hines et al. 2003). Its product, flavin-containing monooxygenase 1 (FMO1), oxidizes the thioether sulfur of some OP and carbamate insecticides (Hajjar and Hodgson 1980), and for some substrates (e.g., fenthion; Furnes and Schlenk 2004) the resulting sulfoxide is a weaker AChE inhibitor than is its parent compound. FMO1 does not appear to oxidize other sulfur atoms in OP insecticides (Hajjar and Hodgson 1980) (activate the parent compound to its oxon). Thus, our results are consistent with the possibility that children with a reduced ability to metabolize OP and perhaps carbamate insecticides might be at increased risk of CBT when sufficiently exposed. The apparent specificity of the results to AChE inhibitors is interesting but in part reflects our selection of polymorphisms. Also, even if our results suggest a biological impact of the SNPs and insecticides, it is unknown whether this is a result of AChE inhibition per se or to some other effect of AChE-inhibiting insecticides used residentially during the study period. For example, chlorpyrifos and diazinon induce neurotoxic effects in neonatal rats, even when administered at levels insufficient to inhibit AChE (Slotkin et al. 2008).

The consistency of results across the three SNPs for which we observed an interaction with childhood insecticide exposure is compelling but nevertheless could represent chance associations. Our results were based on modest numbers, and these SNPs have not been studied in independent samples of brain tumor patients, making the probability of false positives high (Wacholder et al. 2004). The interaction involving *FMO1*_C–9536A_ must be interpreted especially cautiously. Whether the net effect of FMO1 would be protective may depend on the insecticide (Buronfosse et al. 1995; Furnes and Schlenk 2004; Levi and Hodgson 1988). Although children are exposed to FMO1 insecticide substrates, including disulfoton used residentially outdoors, we have not identified an FMO1-metabolized insecticide registered for residential use indoors. The interaction with home insecticide exposure in childhood is also puzzling because FMO1 enzyme levels in the brain and liver drop substantially after birth (Koukouritaki et al. 2002; Zhang and Cashman 2006). Still, FMO1 is not absent from these sites and is expressed at greater levels in the lung and small intestine (Zhang and Cashman 2006), presumably relevant to inhaled and hand-to-mouth exposure, respectively.

The presence of interactions between genotype and insecticide exposure occurring during childhood, but generally not during pregnancy, deserves further comment. During prenatal development, maternal enzymes serve as a first line of defense against exogenous exposures, and without maternal biospecimens we were unable to directly examine the effect of this. Also, perhaps fetal expression of some enzymes is too low, regardless of genotype, to alter insecticide dose sufficiently to protect the brain; here again, maternal enzymes may be important. Our data do not suggest a lack of effect of insecticide exposure during this potentially sensitive period, but rather a lack of synergism with fetal genotype.

We did not observe interactions for other PON or FMO SNPs. None were in the promoter region of their respective genes. Also, the effect of the *PON1*_Q192R_ amino acid change is dependent on the substrate, and the R isoform may be protective for chlorpyrifos but not diazinon (Davies et al. 1996; Li et al. 2000; Mutch et al. 2007). FMO1 metabolizes insecticides better than FMO3 (Furnes and Schlenk 2005; Leoni et al. 2008; Usmani et al. 2004). Perhaps more important, given our results for *FMO3*_E158K_, this coding region SNP is in linkage disequilibrium with promoter region polymorphisms that confer opposing effects on FMO3 enzyme activity (Phillips and Shephard 2008).

The childhood insecticide–*PON1*_C–108T_ interaction was confined to children < 3 years of age. This polymorphism has a greater effect on PON1 levels in neonates than in adults (Chen et al. 2003), and adult levels are reached before 3 years of age (Cole et al. 2003). In addition, by this age diet is the main source of chlorpyrifos (Buck et al. 2001; Clayton et al. 2003), so in older children dietary exposure to chlorpyrifos and diazinon may have overwhelmed any interaction between *PON1*_C–108T_ and residential exposure.

Since the time when the children in our study may have been exposed to home insecticides, chlorpyrifos and diazinon have been phased out of residential use in the United States. Nonetheless, children remain exposed to these and other AChE inhibitors not only via the diet but also potentially via drift from use in agricultural areas, on golf courses, and for mosquito control. In the home, OP and carbamate insecticides remain, for example, in topical treatments for lice (malathion) and flea collars (tetrachlorvinphos, carbaryl, propoxur). Therefore, the present study may have had an increased ability to observe the reported interactions because of the greater residential use of AChE inhibitors, yet our results remain relevant.

Another strength of our study is the use of archived DBS, available for participants regardless of survival status. This makes it unlikely that a relationship between genotype and responsiveness to treatment could underlie the observed interactions. Other opportunities for selection bias were present, including during specimen collection (Searles Nielsen et al. 2008). Although it is therefore difficult to rule out bias in main effects, gene–environment interactions are generally unaffected by selection bias (Morimoto et al. 2003). Further, despite the potential for differential reporting of past exposures, this more likely attenuated than caused the interactions we report (Garcia-Closas et al. 1999).

To date, this is the largest study of CBT and genetic polymorphisms. Studies with more participants are needed to clarify the reported associations. Inclusion of additional polymorphisms in *FMO3* and *BCHE*, especially those in the promoter region, would be worthwhile. These have been less studied than coding region polymorphisms in relation to cancer, but they appeared to be critical here. Objective measurement of specific insecticides in environmental or biological specimens, and detailed interview data on the timing of exposure (e.g., during spermatogenesis, by pregnancy trimester, and by childhood age) also would be important. Although our results most strongly indicated the importance of exposures during early childhood, it is likely that other periods are also important, notably prenatal development. In studies that do consider exposures before birth, it would be useful to assess parents’ genotypes and levels of selected enzymes, including PON1 and FMO1 that are relatively stable over time in adults.

## Figures and Tables

**Table 1:** Functional effect and hypothesized “high-risk” alleles in genetic pesticide metabolism polymorphisms.

Enzyme	Function	Expression	Polymorphism	Hypothesized “high-risk” allele
Paraoxonase (PON1)	Hydrolyzes environmentally and CYP-activated intermediates (AChE-inhibiting oxons) of selected OP insecticides (e.g., chlorpyrifos, diazinon, parathion) such that they cannot inhibit AChE	In blood and liver; expressed during the fetal period; increases with gestational age and after birth; adult levels reached at 6–25 months of age	*PON1*_C–108T_ (rs705379), adjacent to Sp1 binding site in the promoter region	T: Reduced PON1 levels in neonates (63% lower in those with 2 vs. 0 variants) (Chen et al. 2003) and adults (Brophy et al. 2001; Leviev and James 2000)
			*PON1*_Q192R_ (rs662), amino acid substitution near the catalytic center	Q: Reduced brain protection in mice (Li et al. 2000), metabolism in human liver microsomes (Mutch et al. 2007), and metabolism *in vitro* (Davies et al. 1996) for chlorpyrifos but not diazinon
			*PON1*_L55M_ (rs854560), amino acid substitution	M: Less stable PON1 (Leviev et al. 2001)
Butyrylcholinesterase (BuChE)	Sequesters all OP and carbamate insecticides through stoichiometric (1:1) binding such that they cannot inhibit AChE	In brain, blood, lung, intestine, and embryonic tissues	*BCHE*_A539T_ (rs1803274), amino acid substitution	T (“K variant”): 30–40% reduction in BuChE activity (Babaoglu et al. 2004; Bartels et al. 1992; Maetzler et al. 2009)
Flavin-containing monooxygenase (FMO1 and FMO3)	Metabolize some OP insecticides (e.g., phorate, terbufos, fenthion, disulfoton, fonofos) and some carbamate insecticides (e.g., aldicarb and methiocarb); oxidizes the thioether sulfur to form a sulfoxide; does not form oxons	FMO1: highest in the embryo during extensive brain development, and high throughout the fetal period; decreases in the liver and brain after birth; also present in the small intestine and lung (at levels greater than liver after birth)	*FMO1*_C–9536A_ (rs12720462), in a promoter region YY1 binding site	A (*6): Eliminates YY1 binding, 2- to 3-fold loss of promoter activity (Hines et al. 2003)
	Metabolize ethylene thiourea, metabolite of ethylene bisdithiocarbamate fungicides (e.g., maneb, mancozeb, zineb, metiram)	FMO3: Not present in the postembryonic fetal period; in brain by 1–2 years of age	*FMO3*_E158K_ (rs2266782), amino acid substitution	K: One-third activity (Lattard et al. 2003)
Aldehyde dehydrogenase 3A1 (ALDH3A1)	Metabolizes a product of permethrin (pyrethroid insecticide)	In brain, stomach, and lung	*ALDH3A1*_S134A_ (rs887241), amino acid substitution	A: May reduce enzyme activity (Satomichi et al. 2000)
Glutathione *S*-transferase theta 1 (GSTT1)	Metabolizes OC insecticides (DDT, lindane), OP insecticides (methylparathion, EPN), triazine herbicides (atrazine), and chloroacetanilide herbicides (alochlor)	In brain, liver (including during the fetal period), lung, small intestine, and blood (erythrocytes)	*GSTT1* null	*0 (null): No GSTT1 enzyme

Abbreviations: DDT, dichlorodiphenyltrichloroethane; EPN, ethyl *p*-nitrophenyl phenylphosphonothioate.

**Table 2:** Characteristics of children with and without brain tumors, West Coast Childhood Brain Tumor Study, children with genotyping data and born in California or Washington State in 1978–1990 [no. (%)].^a^

Characteristic	Cases (*n* = 201)	Controls (*n* = 285)
Study center		
Los Angeles	110 (55)	99 (35)
San Francisco	25 (12)	50 (18)
Seattle	66 (33)	136 (48)
Birth year		
1978–1984	99 (49)	141 (49)
1985–1990	102 (51)	144 (51)
Age (years)		
< 5	167 (83)	222 (78)
5–10	34 (17)	63 (22)
Child’s race/ethnicity^b^		
White	105 (54)	192 (68)
Hispanic	62 (32)	61 (22)
African American	14 (7)	13 (5)
Asian/other	15 (8)	17 (6)
Male	121 (60)	168 (59)
Brain tumor in first-degree relative, or personal/family history of Li-Fraumeni syndrome, neurofibromatosis, or tuberous sclerosis	3 (1)	3 (1)
Farm residence during pregnancy/childhood	9 (4)	5 (2)
Maternal agricultural occupation in pregnancy Chemical treatment of home for insect pests^c^	4 (2)	4 (1)
During pregnancy^d^	55 (27)	60 (21)
During childhood, up to diagnosis/reference^e^	46 (23)	94 (33)
Insecticides for home, yard, garden, pets, or lice^f^		
During pregnancy^d^	61 (78)	48 (77)
Fleas or ticks	33 (41)	19 (28)
Nuisance pests^g^	43 (57)	39 (60)
During childhood^e^	60 (77)	46 (70)
Fleas or ticks	33 (41)	22 (32)
Nuisance pests^g^	41 (51)	40 (59)

a All study participants for whom a usable DBS was obtained from newborn screening archives in California or Washington State.

b African American: either parent African American; Hispanic: either parent Hispanic and neither parent African American; white: both parents non-Hispanic white; percentages exclude five cases and two controls with non-Hispanic white mothers and for whom paternal race was unknown.

c Termites, fleas, ants, cockroaches, silverfish, or other pests; percentages exclude participants for whom prenatal (one control) or childhood (one case, one control) insecticide exposure was unknown.

d From 1 month before conception until birth.

e Between birth and diagnosis (cases) or comparable reference date (controls).

f Based on children also participating in a pesticide follow-up study in Los Angeles only (Pogoda and Preston-Martin 1997) and with respective exposure data (76–80 cases and 65–68 controls).

g Ants, cockroaches, and other nuisance pests; does not include termites or fleas.

**Table 3:** Risk of CBT and functional pesticide metabolism polymorphisms and *PON2*, overall and by home insecticide treatment, West Coast Childhood Brain Tumor Study [OR (95% CI)].

		Chemical treatment of the home for insect pests^a^		
Pesticide metabolism polymorphism	All children	Never (116 ca/162 co)^b^,^d^	Ever in pregnancy (53 ca/60 co)^b^,^e^	Ever in childhood (46 ca/94 co)^b^,^f^
	(196 ca/283 co)^b^,^c^			
*PON1*_C–108T_	0.9 (0.7–1.2)	0.7 (0.5–1.0)	1.2 (0.7–2.0)	1.8 (1.1–3.0)*
*PON1*_Q192R_	1.0 (0.7–1.3)	1.0 (0.7–1.4)	0.8 (0.5–1.4)	0.9 (0.5–1.6)
*PON1*_L55M_	1.0 (0.8–1.3)	1.1 (0.8–1.6)	0.7 (0.4–1.3)	1.0 (0.6–1.7)
*PON2*_S311C_	0.9 (0.7–1.2)	0.9 (0.6–1.3)	0.6 (0.3–1.3)	1.1 (0.6–1.9)
*BCHE*_A539T_	0.7 (0.5–1.0)*	0.6 (0.4–1.0)*	0.9 (0.4–1.9)	1.3 (0.6–2.8)
*FMO1*_C–9536A_	1.1 (0.7–1.6)	1.0 (0.6–1.6)	0.9 (0.4–2.0)	2.7 (1.2–5.9)*
*FMO3*_E158K_	1.2 (0.9–1.7)	1.4 (1.0–2.0)	1.0 (0.5–1.8)	1.1 (0.7–2.0)
*ALDH3A1*_S134A_	1.1 (0.8–1.4)	1.1 (0.8–1.6)	1.6 (0.9–2.8)	1.0 (0.6–1.7)
*GSTT1* (null)	0.8 (0.5–1.3)	1.0 (0.5–2.0)	0.3 (0.1–1.0)	0.4 (0.1–1.3)

Abbreviations: ca, cases; co, control.

a Based on maternal report of chemical treatment of the home for termites, fleas, ants, cockroaches, silverfish, or “other” pests by a professional, the mother, or someone else.

b OR and 95% CI, for GSTT1 null versus non-null or for all other polymorphisms per “high-risk” allele [*PON1*_–108T_, *PON1*_192Q_, *PON1*_55M_, *BCHE*_539T_, *FMO1*_–9536A_ (*6), *FMO3*_158K_, *ALDH3A1*_134A_; see Table 1], or *PON2*_311C_, adjusted for race/ethnicity (African American, Hispanic, non-Hispanic white, Asian/other), study center, sex, and age at diagnosis/reference (continuous).

c Excludes five cases and two controls with unknown race/ethnicity.

d No chemical treatment of the home for insect pests any time between 1 month before conception and diagnosis/reference; excludes three cases and two controls with unknown race/ethnicity.

e Chemical treatment of the home for insect pests any time from 1 month before conception and birth; excludes two cases with unknown race/ethnicity.

f Chemical treatment of the home for insect pests any time between birth and diagnosis/reference; excludes two cases with unknown race/ethnicity.

* *p* < 0.05.

**Table 4:** Risk of CBT and *PON1*_C–108T_, *FMO1*_C–9536A_, and *BCHE*_A539T_, by home insecticide treatment during childhood and child’s race/ethnicity,^a^ West Coast Childhood Brain Tumor Study [OR (95% CI)].^b^

	Chemical treatment of the home during childhood for insect pests^c^	
Pesticide metabolism polymorphism	Yes	No
*PON1*_C–108T_ (per T allele)		
Non-Hispanic white (105 ca/191 co)^d^	1.5 (0.8–2.8)	0.8 (0.6–1.3)
Hispanic (62 ca/61 co)^e^	1.6 (0.5–5.7)	0.6 (0.3–1.0)

*FMO1*_C–9536A_ (AA/AC vs. CC)		
Non-Hispanic white (105 ca/191 co)^d^	3.0 (1.1–8.3)	0.7 (0.3–1.7)
Hispanic (62 ca/61 co)^e^	3.3 (0.5–23.8)	0.6 (0.3–1.3)

*BCHE*_A539T_ (TT/AT vs. AA)		
Non-Hispanic white (105 ca/191 co)^d^	1.2 (0.5–3.1)	0.5 (0.3–1.0)
Hispanic (62 ca/61 co)^e^	2.9 (0.2–34.3)	0.5 (0.2–1.2)

Abbreviations: ca, cases; co, control.

a Hispanic: neither parent African American and one or both parents Hispanic; non-Hispanic white: both parents non-Hispanic white.

b Data are OR (95% CI) obtained from a single model for each racial/ethnic group containing variables for genotype, childhood exposure (any vs. none), gene–exposure product term, study center, sex, and age at diagnosis/reference (continuous).

c Based on maternal report, chemical treatment of the home for termites, fleas, ants, cockroaches, silverfish or “other” pests by a professional, the mother, or someone else, between birth and diagnosis/reference.

d Data are for 28 cases/73 controls exposed during childhood and 77 cases/118 controls unexposed during childhood (including 9 cases/11 controls with prenatal exposure); excludes one control without exposure information.

e Data are for 12 cases/12 controls exposed during childhood and 50 cases/49 controls unexposed during childhood (including 14 cases/17 controls with prenatal exposure).
